# Pneumatosis Intestinalis, Pneumoperitoneum, and Ascites Secondary to Scleroderma: A Case Report

**DOI:** 10.7759/cureus.27200

**Published:** 2022-07-24

**Authors:** Sean M McCormack, Mary Zahnle, Rangin Haji Rahman, Alvin D Sanhueza-Martinez, Marium Qaisar, Anila Punjwani, Rahul Varghese, Frederick Tiesenga

**Affiliations:** 1 General Surgery, Saint James School of Medicine, Park Ridge, USA; 2 General Surgery, Avalon University School of Medicine, Willemstad, CUW; 3 General Surgery, West Suburban Medical Center, Chicago, USA

**Keywords:** conservative approach, abdominal paracentesis, massive ascites, massive pneumoperitoneum, pneumatosis cystoides intestinalis, pneumatosis intestinalis, systemic scleroderma

## Abstract

Pneumatosis intestinalis (PI), pneumoperitoneum, and ascites are radiographic findings that may be incidental or associated with severe bowel compromise. Asymptomatic patients with benign PI, pneumoperitoneum, or ascites are often observed or treated conservatively. However, these findings are concerning in symptomatic patients and often require surgical consultation and urgent surgical intervention Approximately 15% of PI cases are idiopathic, and 85% are secondary due to an underlying pathology including but not limited to pulmonary disease, autoimmune disease, drug-induced sources, gastrointestinal disease, infectious sources, and iatrogenic sources. A management plan for PI proves challenging to create when the pathogenesis is poorly understood and the presenting clinical picture varies. Reported is a case of a 51-year-old female with severe abdominal pain, PI, pneumoperitoneum, and ascites. Managing a patient presenting this way with surgical intervention is a viable option; however, this patient’s management was successful using a conservative approach.

## Introduction

Radiologic findings of pneumatosis intestinalis (PI), pneumoperitoneum, and ascites suggest acute bowel perforation and often require emergent surgery [1]. Our patient presented with all these radiological findings upon admission. The underlying etiology of these radiological findings can be multifactorial, and it is essential to treat according to the underlying causes. The severity of the condition can range from a benign condition that requires no specific therapy to an acute/life-threatening issue requiring emergency surgery [2]. We will look at the underlying cause of the radiologic findings in this patient and how an emergency surgical laparoscopic intervention was determined unnecessary.

Reported is a case of a 51-year-old female with painful abdominal swelling, pneumoperitoneum, ascites, constipation, and PI for which the etiology was ultimately determined to be secondary due to her past medical history of scleroderma and mixed connective tissue disease. Scleroderma, also known as systemic sclerosis (SSc), has been recognized as an etiology of PI and pneumoperitoneum since 1978. A previous search of both PubMed and the Japanese Medical Abstracts Society demonstrates a handful of cases of SSc complicated by PI [3]. SSc has a low prevalence in Europe and North America, with 7.2-33.9 cases per 100,000 individuals, with an annual incidence rate of 0.6-2.3 cases per 100,000 individuals [4]. This patient has a noteworthy case of PI given the particular autoimmune etiology, which warrants further discussion.

## Case presentation

A 51-year-old female presented to the emergency department with complaints of abdominal fullness, constipation, and nausea with non-bloody, non-bilious emesis. She denied fever, chills, cough, chest pain, or shortness of breath. The patient is a smoker with a past medical history notable for scleroderma, mixed connective tissue disease, protein-losing enteropathy, PI, Raynaud’s disease, heart failure with reduced ejection fraction, deep venous thrombosis (DVT)/pulmonary embolism (PE), chronic ascites, hypertension, hyperlipidemia, asthma, and malnutrition. The patient receives monthly paracentesis at an external facility for chronic ascites. Her surgical history includes prior exploratory laparotomy with no acute findings on exploration other than ascites. Patient home medications include Lovenox for DVT/PE prophylaxis and total parenteral nutrition (TPN) through a tunneled central venous catheter for protein-losing enteropathy/malabsorption syndrome.

Upon arrival, the patient was afebrile with stable vital signs, with a heart rate of 65 BPM, a respiratory rate of 20 BPM, a blood pressure of 137/90 mmHg, and an SPO_2_ of 100% on room air. On examination, the abdomen was distended but non-tender to palpation. Blood and urine laboratory studies taken on the day of admission and the following day showed normal to low total white cell count (6.8 g/L, 4.0 g/L), low hemoglobin (10.8 g/dL, 10.9 g/dL), and hematocrit (34.0%, 35.3%), low potassium (3.3 mmol/L, 3.0 mmol/L), elevated chloride (110 mmol/L, 111 mmol/L) (Table 1). In the emergency room, the patient refused abdominal decompression with a nasogastric tube as this intervention had failed to relieve her symptoms in the past. The patient was admitted to the hospital internal medicine services with a consult request sent to the surgical team.

**Table 1:** Laboratory values over the course of hospital admission. UA - Urinalysis

Date	Day of admission	Day of discharge
Leukocyte count (/mm^3)	6.8	4.0 (L)
Erythrocyte Count (million/mm^3)	4.27	4.41
Hemoglobin, blood (g/dL)	10.8 (L)	10.9 (L)
Hematocrit (%)	34.0 (L)	35.3 (L)
Mean corpuscular volume (µm^3)	80	80
Mean corpuscular hemoglobin (pg/cell)	25.3 (L)	24.7 (L)
Mean corpuscular hemoglobin concentration (% Hb/cell)	31.8	30.9
Prothrombin time (seconds)	10.1	
International Normalized Ratio	1	
Sodium Level (mEq/L)	140	141
Potassium Level (mEq/L)	3.3 (L)	3.0 (L)
Chloride Level (mEq/L)	110 (H)	111 (H)
Carbon dioxide (mm Hg)	21 (L)	24
Alkaline phosphatase (U/L)	70	68
Aspartate aminotransferase (U/L)	14	12
Alanine aminotransferase (U/L)	14	11
Blood urea nitrogen (mg/dL)	13	12
Glucose (mg/dL)	87	75
Creatinine Level (mg/dL)	0.59 (L)	0.58 (L)
Calcium Level (mg/dL)	8.7	8.1 (L)
Phosphorus Level (mg/dL)		3.9
Protein Total (g/dL)	7.6	6.8
Albumin Level (g/dL)	3.2 (L)	2.9 (L)
Bilirubin Total (mg/dL)	0.3	0.4
Anion Gap (mEq/L)	9	6 (L)
Magnesium Level (mg/dL)		1.6
Troponin-I (ng/L)	<0.01	
UA Color		Yellow
UA pH		6
UA Specific Gravity		>=1.030
UA Glucose		Negative
UA Bilirubin		Negative
UA Ketones		Negative
UA Blood		Trace (A)
UA Protein		Trace (A)
UA Urobilinogen (mg/dL)		0.2
UA Nitrite		Negative
UA Leukocyte Esterase		Negative
COVID-19 Polymerase chain reaction	Negative	

**Figure 1:**
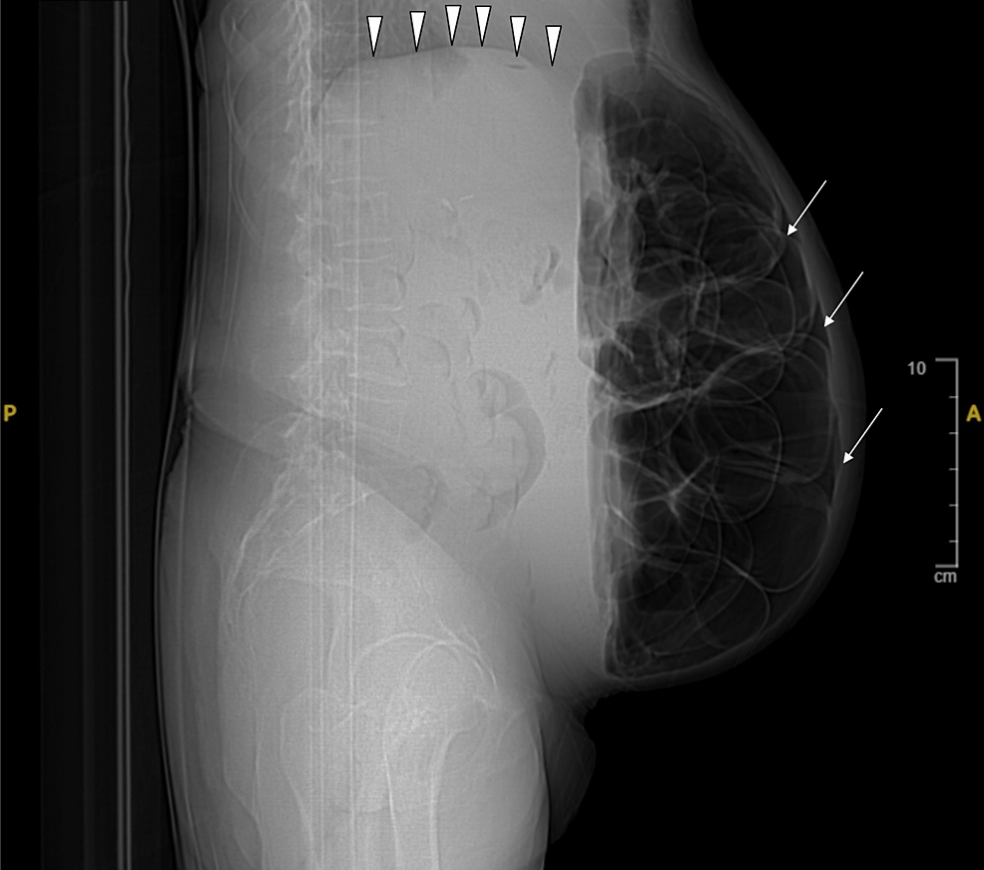
X-ray of the abdomen and pelvis without contrast, supine position, lateral view displaying diffuse ascites (white arrowheads) and pneumoperitoneum (white arrows).

**Figure 2:**
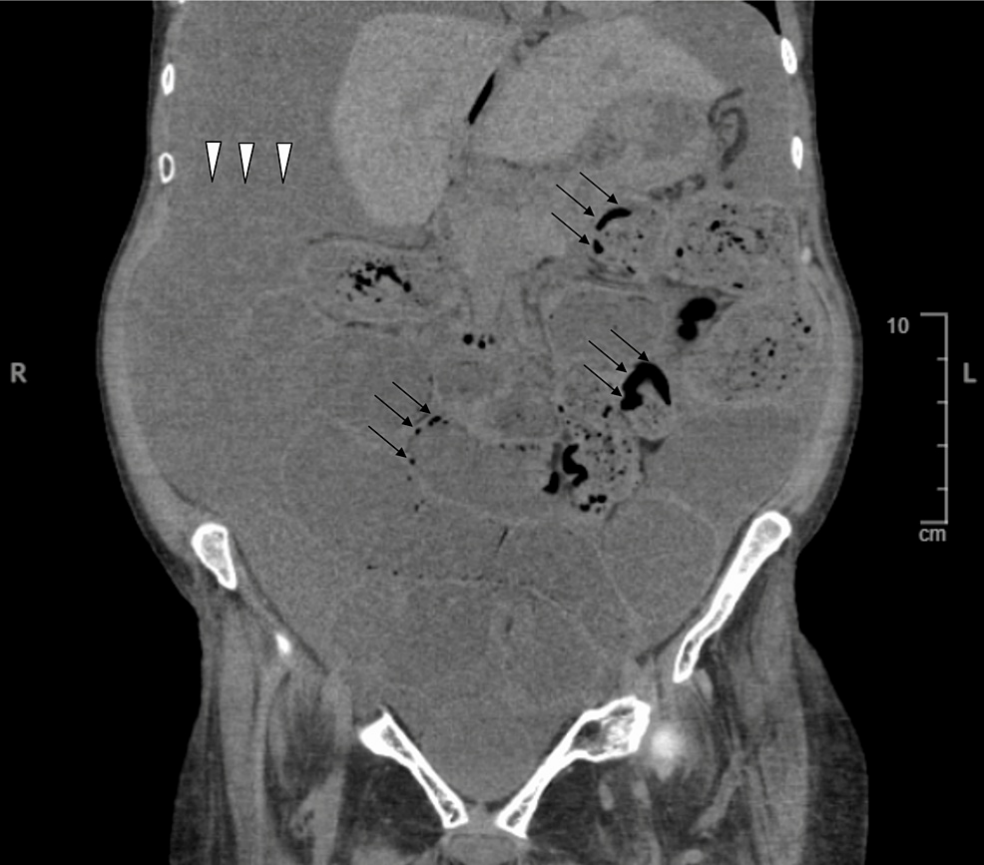
CT of the abdomen and pelvis without contrast, supine position, coronal view displaying diffuse ascites (white arrowheads) and PI (black arrows). PI - pneumatosis intestinalis

**Figure 3:**
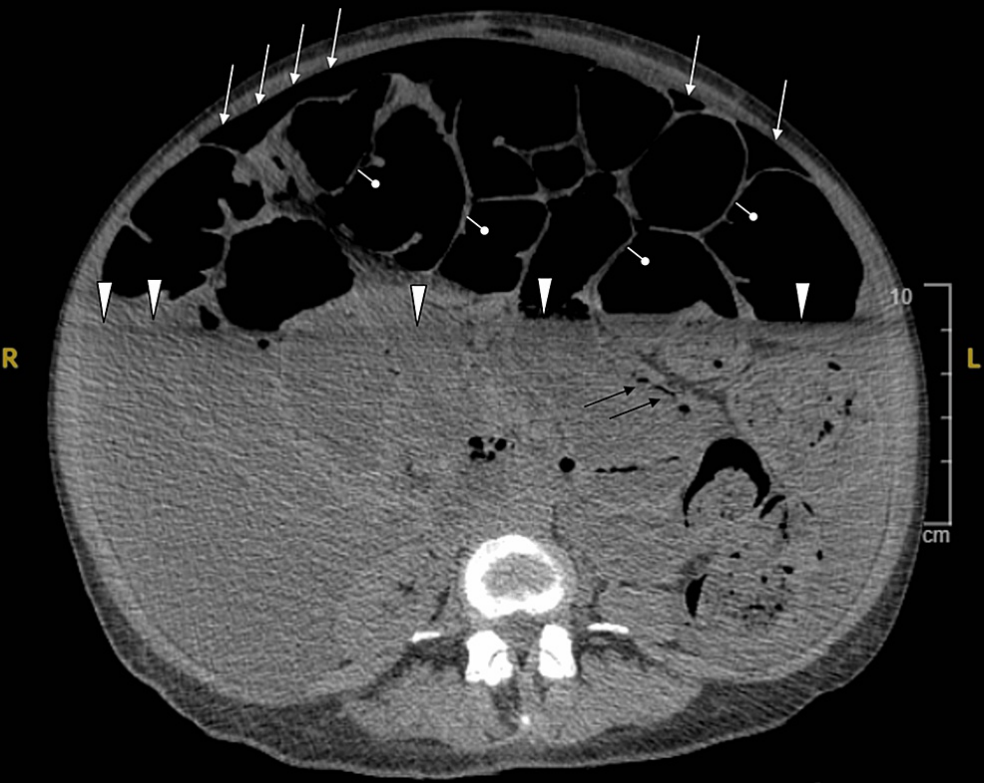
CT of the abdomen without contrast, supine position, displaying PI (black arrows), diffuse ascites (white arrowheads), and PI (black arrows), atrophy of intestinal villi (pinheads), and pneumoperitoneum (white arrows). PI - pneumatosis intestinalis

**Figure 4:**
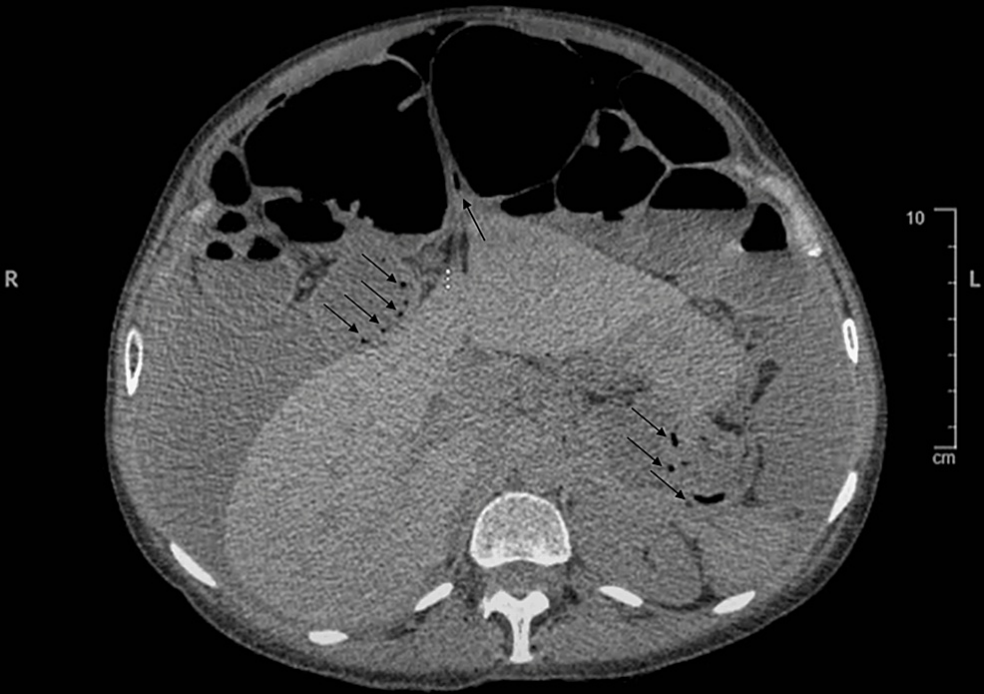
CT of the abdomen without contrast, supine position, displaying PI (black arrows). PI - pneumatosis intestinalis

Findings of a chest and abdominal x-ray included severe gaseous bowel dilation throughout the abdomen and pelvis with multiple air-fluid levels and pneumoperitoneum (Figure 1). Computed tomography (CT) (Figures 2-4), ordered due to concern of bowel obstruction with perforation, showed pneumoperitoneum with no conspicuous source, and large free fluid throughout the abdomen and pelvis. Severe fluid and gaseous dilation of the small bowel, right colon, and transverse colon was seen, without a high-grade transition point. Dilation without a transition point favors partial bowel obstruction or ileus, and both were considered. Other radiological findings noted were PI and moderate stool in the left colon.

Upon examination, by the surgical team, the patient reported being pain free. Due to the patient’s previous surgical history of exploratory laparoscopy without any acute findings, the decision was made to proceed with conservative treatment to which the patient agreed.

Compared to a previous CT scan, no acute progression was noted, and no surgery was needed. The ascites was treated via paracentesis, and its etiology is unknown but likely to be caused by the underlying autoimmune disease. While waiting for paracentesis, an episode of flatulence relieved abdominal pain, nausea, and vomiting. Subsequent paracentesis yielded the removal of 5,300 mL of clear yellow fluid. The fluid was described as a transudate. Laboratory analysis was not formally performed as the ascites was previously documented, and known to be recurring. The patient was discharged later that day. The treatment plan was formulated to treat symptoms conservatively and continue TPN at home. The patient was advised to continue with monthly follow ups for paracentesis as needed.

## Discussion

Pathophysiology

**Figure 5:**
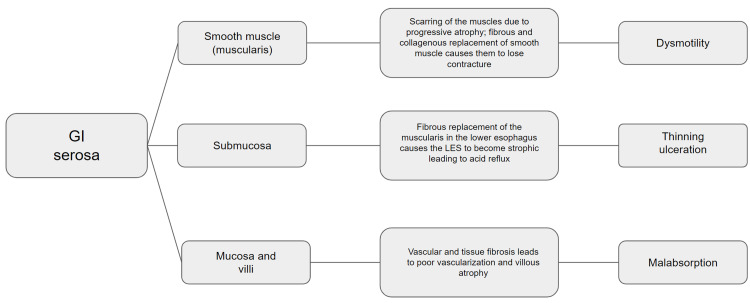
GI pathophysiology of scleroderma

Systemic scleroderma (SSc) is a complex systemic autoimmune disease. The pathophysiology is not entirely understood and is likely multifactorial. The etiology is unknown, and multiple organs are involved, resulting in both adaptive and innate immune irregularities. It targets the vasculature and the extracellular matrix synthesis through activated fibroblasts in various end organs. Models have shown that increased TGF-β is linked to fibrosis. How enhanced fibrosis affects the alimentary tract and results in various outcomes is depicted in Figure 5. Outcomes include dysmotility, thinning/ulceration, and malabsorption [5-7].

PI refers to the presence of gas in the wall of the intestine distal to the stomach. Gas found in the mucosa, submucosa, subserosa, or in all three layers simultaneously is considered PI. PI is a finding most often secondary to an underlying disease process and has only been found to be idiopathic in about 15% of cases [8]. The pathogenesis of PI is not completely understood and is likely multifactorial. Several proposed theories explain the pathophysiology. Theories include a mechanical theory hypothesizing gas from the bowl dissects through the mucosal or the serosal surface at the location of mesenteric blood vessels or breaks in the mucosa. The gas then spreads along the alimentary canal [8,9]. A second theory is that the gas found in the bowel wall comes from gas-forming bacteria. The excess gas in the lumen increases luminal pressure, and gas may be forced directly through the mucosa. The final theory we address is one centered on bowel dysmotility and stasis. Impairment in motility can result in both luminal dilation and pseudo-obstruction. This luminal dilation results in the stretching of mucosa and respective layers of the bowel, allowing for gas dissection [8]. Pseudo-obstruction can also result in bacterial overgrowth that can create additional gas [10].

Discussion

PI is clinical or radiologic findings of gas within the bowel wall. Pneumoperitoneum is the presence of extraluminal gas within the peritoneal cavity. These findings, simultaneously or separately, are often consistent with severe intestinal perforation, which should be in the differential diagnosis [2,3,7]. Intestinal perforation or intestinal compromise is a full-thickness loss of bowel wall integrity. Perforation may result from trauma, instrumentation, inflammation, infection, malignancy, ischemia, necrosis, and obstruction. Complications of perforation are of significant concern and can be life-threatening. Perforation can lead to peritonitis, intraabdominal abscess, bacteremia, sepsis, and multiorgan dysfunction. If bowel perforation is suspected, laparoscopic exploration, peritoneal lavage, and closure of the perforation is the preferred treatment in hemodynamically stable patients. Radiologic findings were concerning for perforation in this patient; however, clinical findings of perforation were not present. This patient had pneumoperitoneum and PI findings with the absence of perforation clinical signs such as elevated white blood cell count, fever, tachycardia, tachypnea, hypotension, and rebound tenderness [2].

The pneumoperitoneum seen is likely related to the underlying autoimmune disease. Pneumoperitoneum can result from PI. The intramural gas accumulation of PI forms thin-walled air-filled cysts. Regardless of the location of the cysts, when they rupture, they cause a pneumoperitoneum, which is generally benign [3].

PI is not a primary disease but is secondary to an underlying cause. Treatment and management must inclusively address this underlying cause. In this patient, SSc was the underlying process causing the PI. In patients without signs of peritonitis or sepsis, a conservative, non-surgical approach is warranted. Conversely, indications for surgical intervention include elevated white blood cell count, presence of portal venous gas on imaging, and clinical signs of sepsis or acidosis, of which none were present in this case [2,8].

The patient’s simultaneous presentation of ileus symptoms was determined to be due to her scleroderma. SSc causes fibrosis of the muscular layer of the intestine responsible for synchronized contractions of the intestine known as peristalsis. Disruption of these muscle fibers due to fibrosis leads to progressive atrophy and collagenous replacement. This furthers the damage seen in the circular smooth muscle layer, with increased GI tract dysmotility [5-7]. Progression of SSc can lead to episodes of pseudo-obstruction. In this case, we saw clinical and radiological findings to support a pseudo-obstruction. The patient reported episodes of non-bloody, non-bilious emesis with recurring nausea and reported her last bowel movement to be four to five days prior to admission. CT scan findings were also supportive: severe gaseous and fluid dilation of the small bowel with the ascending and transverse colon showing no high-grade transition point. There was moderate stool in the descending colon. Finding no high-grade transition point favors pseudo-obstruction over other diagnoses. [2,8,11,12]. Ascites also contributed to increasing obstructive symptoms [13].

Lastly, it is crucial to consider the patient’s history of recurrent ascites. SSc can cause damage to the mucosa and villi of the digestive tract, causing malabsorption, as aptly demonstrated by this patient who receives TPN in hospital and at home for malnutrition and protein-losing enteropathy. The patient had a continuous loss of protein and fluid in the third space. Third space or third spacing refers to the movement of bodily fluid from the blood into the spaces between the cells, leading to the accumulation of fluid within body cavities, intestinal areas, or areas of the body that generally contain little fluid [8,13]. Ultrasound-guided paracentesis harvested 5,300 mL of transudative fluid from the third space. The patient’s past surgical history included monthly paracentesis, indicating that her massive ascites was not due to an acute process but rather her chronic condition. Transudative ascites can occur due to multiple mechanisms. It can be either due to increased capillary osmotic pressure or decreased capillary oncotic pressure. This patient had malnutrition and protein-losing enteropathy, which results in decreased intravascular oncotic pressure. The decreased intravascular oncotic pressure allows fluid to flow from protein-deficient capillaries toward the higher interstitial oncotic pressures of the peritoneal cavity. Documented idiopathic portal hypertension (IPH) occurs in cases of SSc, and subsequent elevated venous osmotic pressure can predispose to ascites. Ascites occur due to increased portal venous pressure and hydrostatic pressure in the hepatic vessels that push fluid out of the intravascular space into the peritoneal cavity [13,14]. There are other chronic conditions that also lead to transudative ascites. Common etiologies are broken up into presinusoidal, sinusoidal, and postsinusoidal. Conditions include but are not limited causes such as cardiac failure, liver disorders and hepatorenal thrombosis. While we cannot rule out all of these conditions causing ascites. This patient had multiple comorbidities including heart failure, the cause of the ascites is likely multifactorial and the treatment is the same. Ascites is treated with repeated large volume paracentesis or insertion of a transjugular intrahepatic portosystemic stent shunt (TIPS). TIPS is an invasive procedure without proven survival benefits [15].

## Conclusions

In conclusion, scleroderma patients presenting with the clinical manifestations and radiographic findings suggestive of PI and pneumoperitoneum can be managed using a conservative approach as the radiologic findings are often secondary to autoimmune disease rather than severe bowel compromise or perforation. This report outlined a case of a 51-year-old female who developed PI with pneumoperitoneum and ascites secondary to scleroderma. Our patient has a surgical history of exploratory laparoscopy without acute findings, and clinical signs were not concerning for a more acute process. Similar cases should proceed cautiously with operative intervention based on the likelihood of bowel compromise. In instances where conservative measures do not resolve the symptoms, we favor diagnostic laparoscopy.
